# Efficacy of glucocorticoids for the treatment of macrolide refractory mycoplasma pneumonia in children: meta-analysis of randomized controlled trials

**DOI:** 10.1186/s12890-019-0990-8

**Published:** 2019-12-18

**Authors:** Hwan Soo Kim, In Suk Sol, Donghe Li, Miyoung Choi, Yun Jung Choi, Kyung Suk Lee, Ju Hee Seo, Yong Ju Lee, Hyeon-Jong Yang, Hyun Hee Kim

**Affiliations:** 1 Department of Pediatrics, College of Medicine, The Catholic University of Korea, Seoul, South Korea; 2 Department of Pediatrics, Hallym University Chuncheon Sacred Heart Hospital, Chuncheon, Korea; 3 Department of Pediatrics, Yonsei University College of Medicine, Seoul, Korea; 4 Division of Health Technology Assessment Research, National Evidence-based Healthcare Collaborating Agency (NECA), Seoul, South Korea; 5 Department of Pediatrics, Seoul National University Hospital, Seoul, Korea; 6 Department of Pediatrics, Hanyang University Guri Hospital, Hanyang University College of Medicine, Guri, South Korea; 7 Department of Pediatrics, Dankook University Hospital, Cheonan, South Korea; 8 Department of Pediatrics, Hallym University Kangnam Sacred Heart Hospital, Hallym University College of Medicine, 1 Singil-ro, Yeongdeungpo-gu, Seoul, 07441 South Korea; 9 Pediatric Allergy and Respiratory Center, Department of Pediatrics, SCH Biomedical Informatics Research Unit, Soonchunhyang University Seoul Hospital, Soonchunhyang University College of Medicine, 59 Daesagwan-ro, Yongsan-gu, Seoul, 04401 South Korea

**Keywords:** Pneumonia, Mycoplasma, Macrolides, Glucocorticoids

## Abstract

**Background:**

*Mycoplasma pneumoniae* is one of the most common pathogens causing community acquired pneumonia in children. Although the rate of macrolide-refractory *Mycoplasma pneumoniae* (MRMP) has increased, systemic glucocorticoids as a treatment option has not been validated yet. The purpose of this study was to assess the efficacy of glucocorticoids add-on in the treatment of MRMP in children through systematic review and meta-analysis.

**Methods:**

**Data sources**

A systematic literature search was conducted using ten electronic bibliographic databases including English, Korean, Chinese and Japanese languages, up to March 8, 2018.

**Study selection**

The study was conducted according to Preferred Reporting Items for Systematic Reviews and Meta-Analyses checklist and selected randomized control trials which compared the efficacy of glucocorticoids add-on to macrolide in the treatment of MRMP in children.

**Data extraction**

Two independent reviewers extracted: primary outcomes as hospital days, fever duration, and change in C-reactive protein (CRP) and main analysis was performed through meta-analysis with random effects model.

**Results:**

Twenty-four unique randomized controlled trials met the inclusion criteria. The mean length of hospital stay in glucocorticoids treatment group was significantly shorter than that in conventional macrolide-treatment group (Weighted mean difference (WMD) = − 4.03 days). The mean length of fever duration was significantly shorter in the glucocorticoid treatment group in comparison with the conventional treatment group (WMD = -3.32 days). Level of CRP after treatment was significantly lower in the glucocorticoid treatment group than that in the conventional treatment group (WMD = -16.03). Sensitivity analysis and subgroup analysis showed no significant improvement in heterogeneity. As limitations of the study, most of the studies included were from a single country and we were unable to control for heterogeneity across interventions, lack of standardized measures, and different time points of assessments across studies.

**Conclusions:**

Glucocorticoid add-on treatment for MRMP can significantly shorten the duration of fever and hospital stay and decrease the level of CRP. These results should be confirmed by adequately powered studies in the future.

## Background

*Mycoplasma pneumoniae* (*M. pneumoniae*) is one of the major pathogens causing community acquired pneumonia and bronchitis in children. Treatment of *M. pneumoniae*-related respiratory infection is based on symptomatic treatment with antibiotics. Macrolides have been used as first line treatment. However, macrolide-refractory *M. pneumoniae* (MRMP) strains are increasing abruptly, particularly in East Asian countries including Korea, Japan, and China [1–3].

Most patients with MRMP do not show improvement of fever when they are treated with macrolides. Some may develop refractory or severe clinical course that requires additional treatment. Treatment for MRMP includes tetracyclines, fluoroquinolones, and systemic glucocorticoids [4].

Secondary antibiotics such as tetracyclines and fluoroquinolones are considered as effective alternatives in the treatment of MRMP. However, they are of limited use due to safety-concerns of teeth discoloration and musculoskeletal toxicity, particularly in children. Glucocorticoids can be also considered as alternative treatment options due to two reason. First, the pathogenesis of *M. pneumoniae* infection is associated with amplified host immune response and virulence of *M. pneumoniae* [5]. Second, adverse effects of glucocorticoids have been well established. Further risk is not likely to be added in the treatment of MRMP. However, previous studies on the effect of glucocorticoids have shown conflicting results [6–8].

The objective of this study was to assess the efficacy of glucocorticoids for treatment of MRMP in children through systematic review and meta-analysis.

## Methods

Systematic Review and Meta-Analysis Protocols (PRISMA-P) 2015 were used for this systematic review and meta-analysis [9]. The Population-Intervention-Comparison-Outcome (PICO) question used for our search strategy was: “Does use of glucocorticoids help improve the outcome of MRMP in children?”

### Search strategy

We performed a systematic search utilizing a protocol designed by two independent medical librarians (D.W.S. and M.L) specifically for this study with 10 electronic databases: PubMed, EMBASE, Cochrane Library, and Core journal (Korean, Japanese, and Chinese Journal) Full-text Database. The search encompassed articles published from January, 1990 to March 8, 2018. We used search terms listed in Additional file 1 to search PubMed, Cochrane, EMBASE, and database of core countries. We imposed no language or publication restrictions.

The first screening was executed by two independent reviewers (H.S.K. and I.S.S.) who evaluated the titles and abstracts obtained from the search. Records were managed using Endnote (version X8; Clarivate Analytics, Philadelphia, PA, USA). From this initial screening, articles that did not focus on glucocorticoid use in MRMP and review articles were immediately excluded. After initial exclusion process, full texts of the remaining articles were reviewed independently by two authors (H.S.K. and I.S.S.) to determine whether any articles met the predetermined eligibility criteria described in the next section. Disagreements between the two reviewers regarding the inclusion or exclusion of particular studies were settled by consultation with a third reviewer (Y.J.L.).

### Eligibility

The following inclusion criteria were applied: (1) randomized control trial (RCT) which compare the efficacy of glucocorticoids add-on to macrolide alone in children with MRMP, (2) MRMP which was diagnosed with serology or polymerase chain reaction, and that refractories were defined clinically, (3) only included children < 18 years of age, and (4) outcome measures with hospital days, fever duration, and level of C-reactive protein (CRP) change. Review articles, published abstracts without full-text publications, and case-study reports with 10 participants or less were excluded. Our search strategy included non-English articles in our initial search results. Non-English articles were then translated and included for evaluation.

### Study selection

Two reviewers (H.S.K. and I.S.S.) independently screened titles and abstracts of the studies identified in our systematic search. Studies focusing on MRMP were included after review of abstracts. Full texts from included studies were reviewed to evaluate for eligibility. Reference lists of selected studies and previous reviews were also examined to determine any relevant publications overlooked by the electronic search. Disagreements between the two reviewers in the selection of particular studies were settled after discussion with a third reviewer (Y.J.L.).

### Risk of bias assessment

Cochrane Collaboration Risk of Bias Tool was used by the two reviewers (H.S.K. and I.S.S.) who independently evaluated the risk of bias in included studies [10]. Risk of bias was determined as hi, low or unclear by evaluating random sequence generation, blinding of participants and personnel, incomplete outcome data, and selective reporting. Disagreements between the two reviewers regarding the risk of bias assessment of particular studies were settled after discussion with a third reviewer (Y.J.L.).

### Data extraction

Two reviewers (H.S.K. and I.S.S.) used a structured form to extract data from each eligible study. Data extracted from each study could be characterized as characteristics of the sample, intervention details, and measurement of outcomes. Disagreements between the two reviewers regarding the data extraction of particular studies were settled by consultation with a third reviewer (Y.J.L.). Primary outcomes of the current study were hospital days, fever duration, and change in CRP.

### Statistical analysis

The estimated mean effect of glucocorticoid add-on treatment on hospital days, fever duration, and change in C-reactive protein (CRP) and the associated 95% confidence intervals (CIs) were extracted or calculated for the 24 studies included in the meta-analysis with Review Manager 5.3 (The Cochrane Collaboration, London, United Kingdom). Random-effects model was used for studies included in the analysis.

Heterogeneity was calculated using I^2^ statistic. The I^2^ statistic threshold should always be interpreted with care. A rough estimate of 25% denotes low heterogeneity while 50% denotes moderate heterogeneity, and 75% denotes high heterogeneity [11]. We conducted sensitivity analyses when heterogeneity was noted. This was performed by removing a study from the analysis to determine changes in I^2^ values and assess which studies play a significant role resulting in heterogeneity [11]. To assess the risk of publication bias, we used funnel plots for visual inspection, and Egger test and trim-and-fill method were performed for statistical identifying. All statistical analyses were performed using R (version 3·3·3) and Review Manager 5.3.

## Results

### Systematic literature search results

**Fig. 1:**
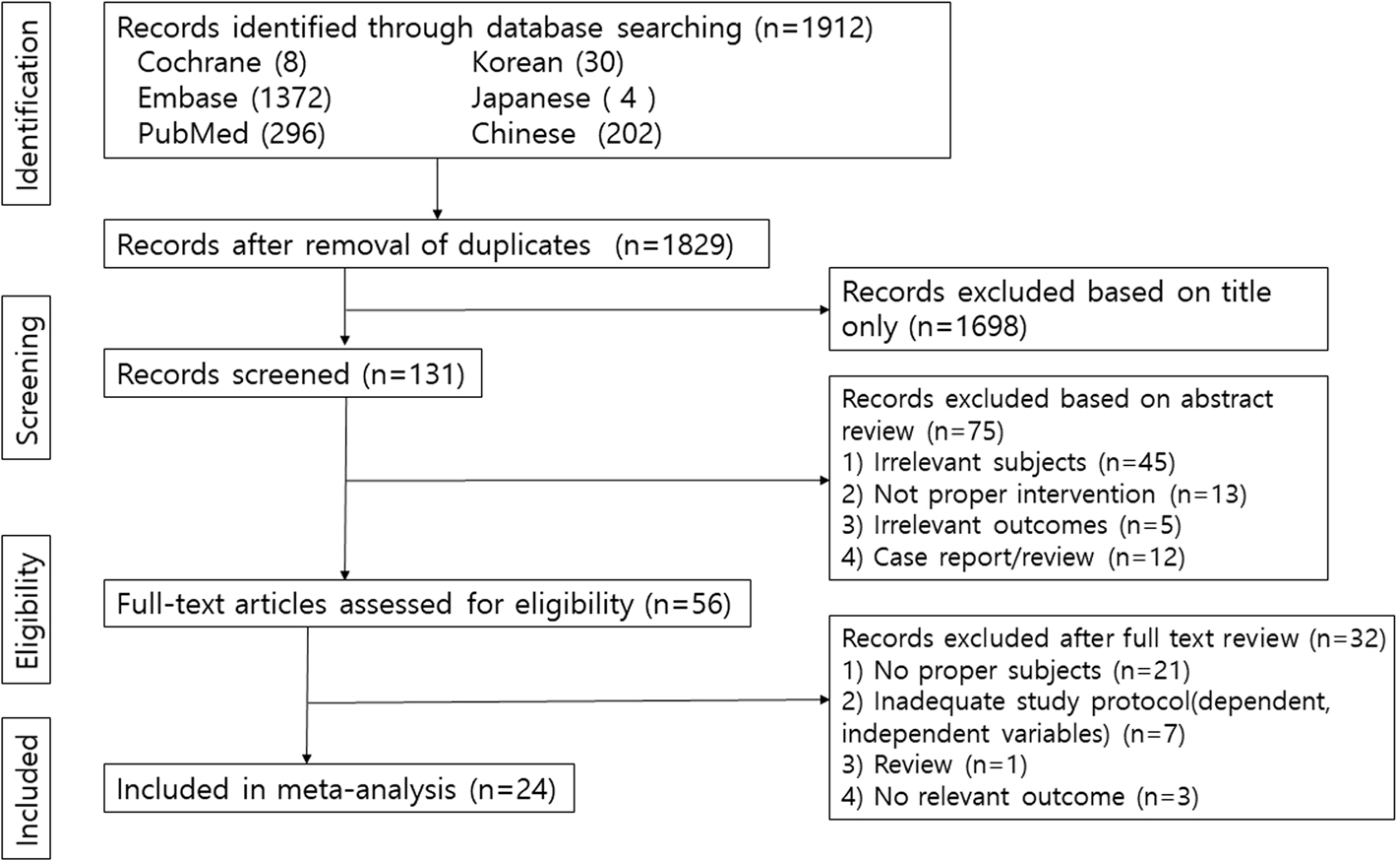
Preferred Reporting Items for Systematic Reviews and Meta-Analyses flowchart

A total of 1829 citations were identified initially. Of these, 1773 studies were discarded after reviewing titles and abstracts, leaving 56 articles for full text review (Fig. 1). A total of 32 studies were excluded after full text review due to no proper subject, inadequate study protocol, review article, or no relevant outcome. A total of 24 studies were included in our systematic review and meta-analysis [12–35]. All studies were RCTs. (Additional file 1 for search strategies for database, Additional file 2 for PRISMA checklist).

### Sample characteristics

Participants of studies enrolled in our meta-analysis was a total of 2365 patients. All these studies examined fever duration. Fifteen studies examined hospital days while 14 studies examined CRP level after treatment (Table 1).

**Table 1:** Characteristics of studies included in the meta-analysis

Study	Characteristics				Intervention		Outcome
	Country	Year	Number of subjects; mean age of experiment group, y	Number of subjects; mean age of control group	Experimental group	Control group	
Fan Xuwei 2015 [18]	China	2012–2015	44; 8.2 ± 2.7	43; 7.2 ± 2.1	MPD (2 mg/kg/d) for 5 consecutive days then received 1 mg/kg/d for 2 days	Oral administration of AZM tablets (10 mg/kg; max. dose 0.5 g) for 1 day then received 5 mg/kg of AZM through day 2–5 (max. dose 0.25 g)	Fever duration, Hospital day, CRP change
Feng Xiaoqiang 2016 [26]	China	2013–2015					Fever duration, Hospital day, Cough duration, Improvement of chest x-ray
Ji Chaoyu 2017 [28]	China	2014–2016	50; 5.1 ± 0.3	50; 4.9 ± 0.5	IV MPD (2 mg/kg/d) for 3 days	Daily IV infusion of AZM (10 mg/kg/d)	Fever duration, Cough duration
Li Ling 2015 [14]	China	2013–2014	53; 6.5 ± 2.1	51; 6.6 ± 1.9	MPD (2 mg/kg/d) for 3–5 days	Erythromycin IV drip for 1 week, then change to AZM IV drip for 3 days, stop for 4 days, then oral AZM tablets for 3 days, then stop for 4 days then oral AZM tablets for 3 days again, with 3rd generation cephalosporin	Fever duration, Hospital days, Cough duration, Change in chest x-ray, CRP change
Li Ming 2015 [15]	China	2013–2014	50; 3.1 ± 0.4	50; 3.2 ± 0.2	IV administration of prednisolone sodium succinate 1–2 mg/kg/d for 3 days, then changed to oral administration of prednisone 1–2 mg/kg/d, then stopped 7–10 days of tapering	Daily IV administration of AZM (10 mg/kg/d) for 3–5 days, then stopped for 3 days. Sequential therapy with daily administration of AZM dry suspension 10 mg/kg/d for 3 days then stopped for 4 days, and repeated for total course of treatment of 1 month	Fever duration, Hospital days, Cough duration, CRP change
Lin Jianqin 2015 [16]	China	2012–2015	42; 6.4 ± 1.2	41; 6.1 ± 1.3	IV MPD 1 mg/kg/time, 2 times/day, for 3 days, then changed to oral administration of MPD, 1 mg/kg/time, 2 times/day	Daily IV AZM 10 mg/kg, for 3–5 days then oral administration of AZM 10 mg/kg/d for 3 days then stop for 4 days. Oral administration was repeated for 2–3 times during course of treatment	Fever duration, Cough duration, Time to normalization of chest x-ray
Lin Yan 2015 [17]	China	2012–2015	45; 6.4 ± 3.2	45; 6.7 ± 3.3	IV infusion of dexamethasone 0.2–0.3 mg/kg/d for 5 days	IV infusion of AZM and gamma globulin	Fever duration, Hospital day, Cough duration, Time to normalization of chest x-ray, CRP change
Liu Chunyan 2017	China	2015–2016	52; 5.8 ± 4.0	52; 5.6 ± 4.2	IV MPD pulse therapy (1–2 mg/kg/d) for 3 days	IV infusion of immunoglobulin 400 mg/kg/d for 2 days; IV infusion of AZM 10 mg/kg/d for 5 days	Fever duration, Time to normalization of chest x-ray
Liu Qing 2016 [22]	China	2013–2015	74;	62;	IV infusion of MPD 2 mg/kg/d was administered until 24 h after defervescence. Oral prednisone was started with 1–2 mg/kg/d then tapered for 7–14 days	IV infusion of AZM 10 mg/kg/d for 5 days then stop 4 days and repeat for 2–3 cycles	Fever duration
Lu Xiaoyun 2017 [29]	China	2014–2015	53; 6.59 ± 1.57	52; 6.80 ± 1.43	IV infusion of MPD 2 mg/kg/d for 5 days	10 mg/kg of oral AZM for 1 day continued by 5 mg/kg of AZM from day 2–5.	Fever duration, Cough duration, Time to normalization of chest x-ray, CRP change
Qiu Haiyan 2017	China	2015–2016	50; 6.91 ± 2.16	50; 6.85 ± 2.10	MPD 1–2 mg/kg/d	IV AZM (10 mg/kg/d) was used until symptom improvement then changed to daily oral AZM suspension 10 mg/kg/d	Fever duration, Cough duration, CRP change
Ren Mingxing 2015	China	2011–2013	33; 8.9 ± 2.4	34; 9.3 ± 3.0	MPD 2 mg/kg/d for 5 days then reduced to 1 mg/kg/d for 2 days	IV infusion of aspartate AZM 10 mg/kg/d for 3 days; daily IV infusion of gamma globulin 1.5 g/kg for 3 days; IV infusion of rifampicin 10 mg/kg/d for 3 days then stopped for 4 days then change to oral administration of AZM 10 mg/kg/d for 3 days then stopped for 4 days. Total duration of treatment was 7 days for one course of treatment and was continued for 3 weeks	Fever duration, Hospital days, CRP change
Shan Li-Shen 2017 [35]	China	2013–2015	52; 7.36 ± 2.33	50; 7.29 ± 3.03	Oral or IV MPD 2 mg/kg/d for 3 days	IV AZM	Fever duration, CRP change, LDH change, D-dimer change
Shao Xiaoli 2011 [12]	China	2008–2010	38; 6.37 ± 2.83	38; 6.87 ± 2.86	Small dose of MPD for 3–4 weeks	Macrolide antibiotics	Fever duration, Hospital days, Cough duration, Chest X-ray change
Tao Xuyun 2015	China	2013–2014	75; 7.4 ± 1.4	75; 7.3 ± 1.3	IV MPD 2 mg/kg/d for 4–5 days then on 5–7 day of treatment, dose increased to 4 mg/kg/d according to patient symptoms. Then reduced to 1 mg/kg/d for 3 days after defervescence.	IV AZM (10 mg/kg/d) for 3 days then stopped for 4 days. Followed by oral AZM for 3 days then stopped for 4 days continued for 3 weeks with ceftazidime	Fever duration, Hospital day, Cough duration, Change in chest X-ray, CRP change
Wang Hao 2016 [24]	China	2013–2015	40; 5.10 ± 1.86	40; 4.86 ± 1.35	4 consecutive days with 2 mg/kg/d of MPD then reduced to 1 mg/kg/d	Daily IV infusion of AZM 10 mg/kg/d for 3 days. Then changed to 5 mg/kg/d of oral AZM, 3 times/day, for 3 days then stopped for 4 days	Fever duration, Hospital day, CRP change
Wen Jianjun 2016 [23]	China		65; 7.1 ± 4.5	65; 7.7 ± 4.5	IV infusion of MPD (2 mg/d, 1–2 times) and reduced as symptoms improved	IV AZM 10 mg/kg/d for 3 days then stopped for 4 days. Changed to oral AZM after symptoms improve	Fever duration, Hospital days
Wu Yourong 2017 [33]	China	2013–2014			MPD 2 mg/kg/d for 3 days. Then changed to 1 mg/kg/d for 2 days	IV infusion of AZM 10 mg/kg/d for 3 days. After 3 consecutive days of treatment, oral AZM (10 mg/kg/d) was administered for 3 days then stopped for 4 days	Fever duration, Hospital days
Xu Jiali 2017	China	2015–2017	60; 6.8 ± 1.6	60; 7.1 ± 2.5	Oral intake of MPD (2 mg/kg/d) for 3–5 days on 2nd day of treatment	Daily oral intake of AZM 10 mg/kg/d for 3 days then stopped for 4 days then repeated for 3–4 times	Fever duration, Hospital days, Cough duration, CRP change
Yang Lijun 2015 [19]	China	2012–2014	20	20	IV administration of MPD (1 mg/kg/d) for 2 weeks	IV infusion of AZM (7–10 mg/kg/d)	Fever duration, Hospital days, Cough duration
Yu Jieming 2017 [32]	China	2014–2015	35; 5.6 ± 2.7	35; 5.7 ± 2.3	IV infusion of MPD (2 mg/kg/d), 2 times/day.	IV infusion of erythromycin 20–30 mg/kg/d, 2 times/d. Change to oral AZM (10 mg/kg/d) after 48 h of defervescence	Fever duration, Cough duration, CRP change
Zhang Xiang 2015 [21]	China	2012–2013	32; 5 ± 2	32; 4 ± 1	IV infusion of MPD (1–2 mg/kg/d) with nebulized budesonide, for 3–5 days; If symptoms don’t improve, oral administration of MPD was given for 3–5 days.	IV infusion of erythromycin 20–30 mg/kg/d, for 2 times/day, for 7 days; followed by oral administration of AZM 10 mg/kg/d (max. dose 0.5 g/d), for 3 days then stopped for 4 days	Fever duration, Hospital days
Zhao Shuqing 2017 [34]	China	2013–2015	29; 5.7 ± 2.4	29; 5.3 ± 2.5	Daily IV MPD 1.5–2.0 mg/kg/d for 3 days, then changed to 1 mg/kg/d and tapering within 1 week	IV AZM 10 mg/kg/d on the 1st day, 5 mg/kg/d from 2nd to 5th day, 5 days as a total treatment course	Fever duration, Cough duration, CRP change
Zheng Xuan 2016 [25]	China	2015–2016	70; 5.5 ± 0.5	70; 5.1 ± 0.6	IV infusion of MPD (2 mg/kg/d) for 3 days	IV infusion of AZM (10 mg/kg/d) for 3 days	Fever duration, Cough duration, CRP change

*Abbreviations*: *AZM* azithromycin, *CRP* C-reactive protein, *IV* intravenous, *LDH* lactate dehydrogenase, *MPD* methylprednisolone

### Fever duration

**Fig. 2:**
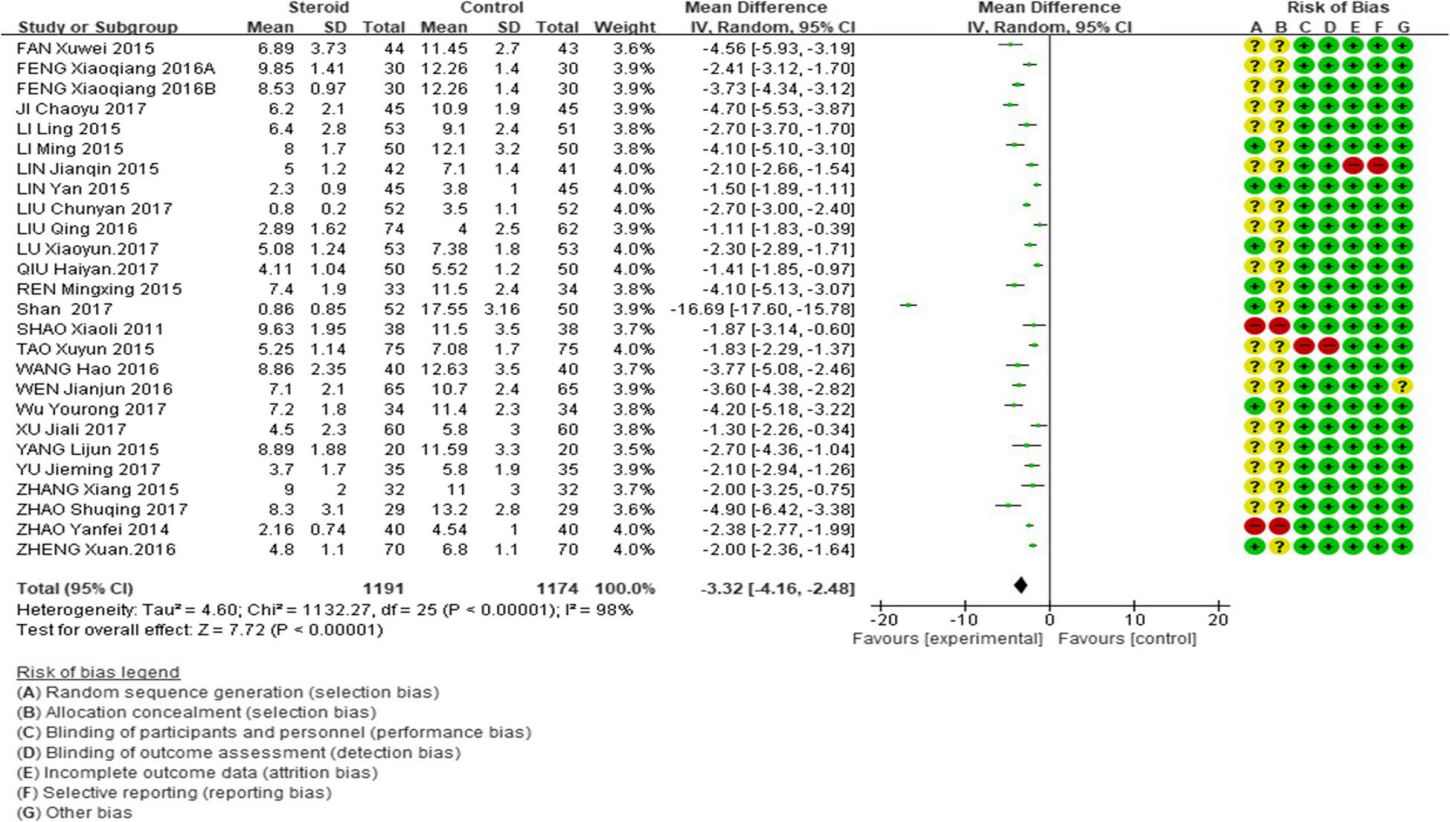
Comparison of fever duration between glucocorticoid therapy and macrolide therapy

The mean length of total duration of fever was significantly shorter in the glucocorticoid add-on group than that in the conventional treatment group (weighted mean difference, WMD = − 3.32, 95%CI: − 4.16 – -2.48, Z = 7.72, *P* < 0.00001). However, there was a high between-study heterogeneity of this effect (I^2^ = 98%; Fig. 2).

### Hospital days

**Fig. 3:**
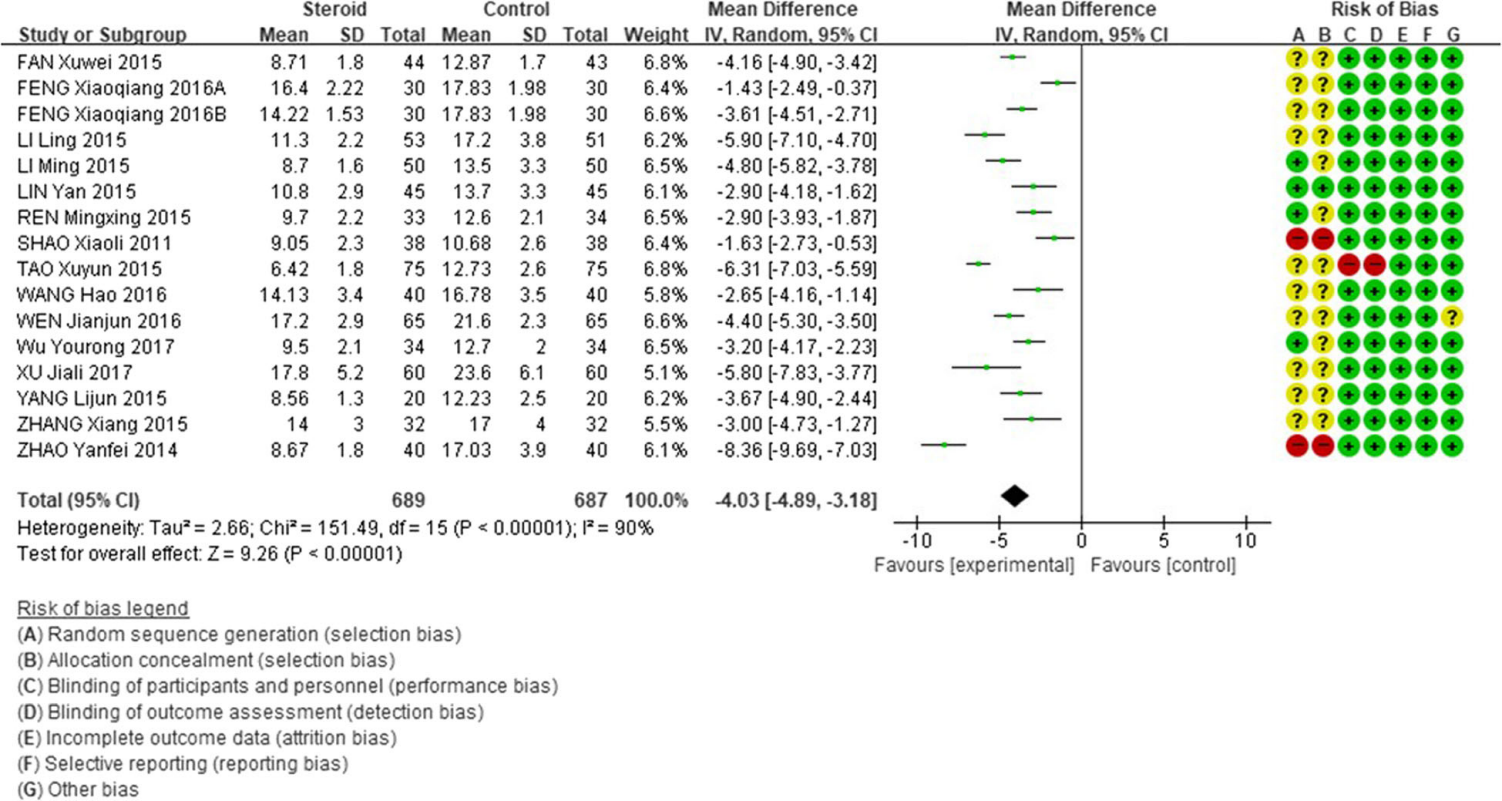
Comparison of hospital days between glucocorticoid therapy and macrolide therapy

The mean length of hospital stays in the glucocorticoid add-on treatment group was significantly shorter than that in the conventional treatment group (WMD = − 4.03, 95% CI: − 4.89 - -3.18, Z = 9.26, *P* < 0.00001). However, there was a high between-study heterogeneity of this effect (I^2^ = 90%; Fig. 3).

### Reduction of CRP level after treatment

**Fig. 4:**
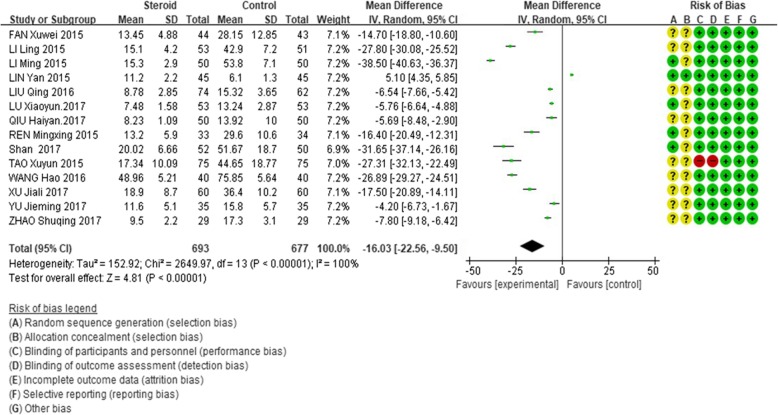
Comparison of C-reactive protein level after treatment between glucocorticoid therapy and macrolide therapy

The level of CRP after treatment was significantly lower in the glucocorticoid add-on treatment group than that in the conventional treatment group (WMD = − 16.03, 95%CI: − 22.56 – -9.50, Z = 4.81, *P* < 0.00001). However, there was a high between-study heterogeneity of this effect (I^2^ = 100%; Fig. 4).

Sensitivity analyses was performed because of high level of heterogeneity. We removed a study from the analysis to determine which studies contributed most significantly to the heterogeneity by determining the changes in I^2^ values. We found that I^2^ values of fever duration, hospital days, and CRP level did not change.

### Subgroup analysis

**Fig. 5:**
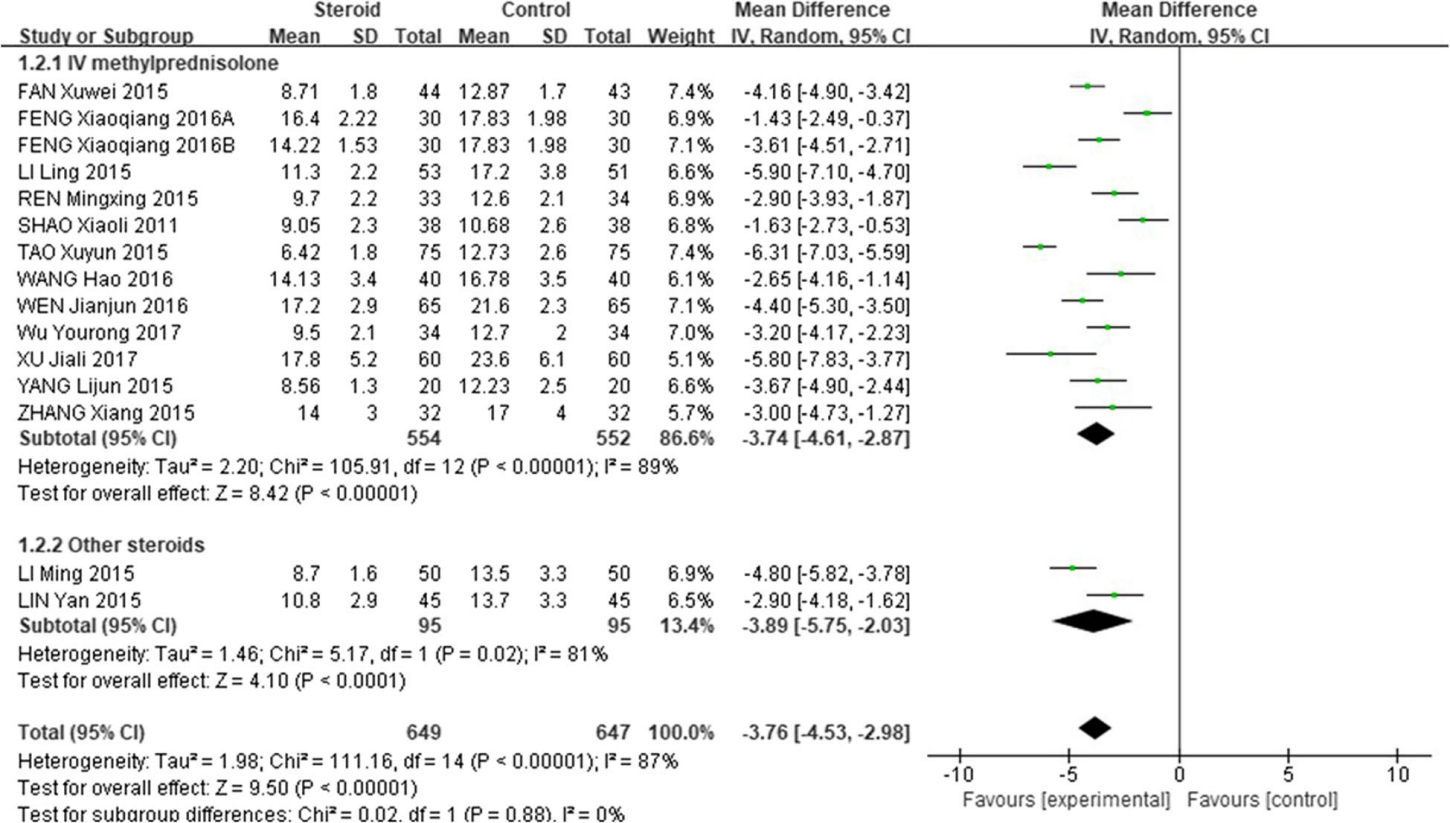
Forest plot for hospital days in subgroup analysis with use of methylprednisolone and other steroids

**Fig. 6:**
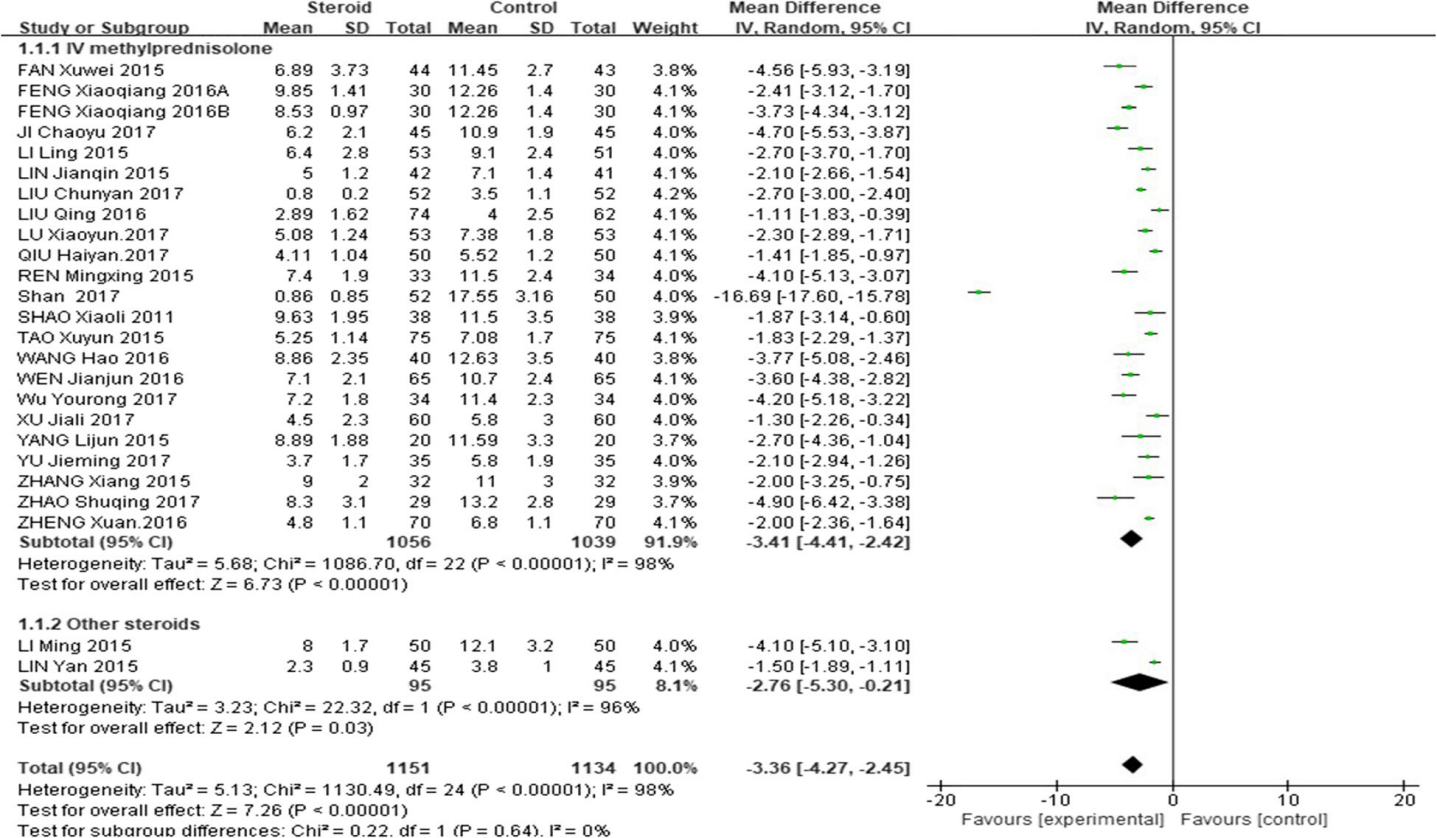
Forest plot for fever duration in subgroup analysis with use of methylprednisolone and other steroids

**Fig. 7:**
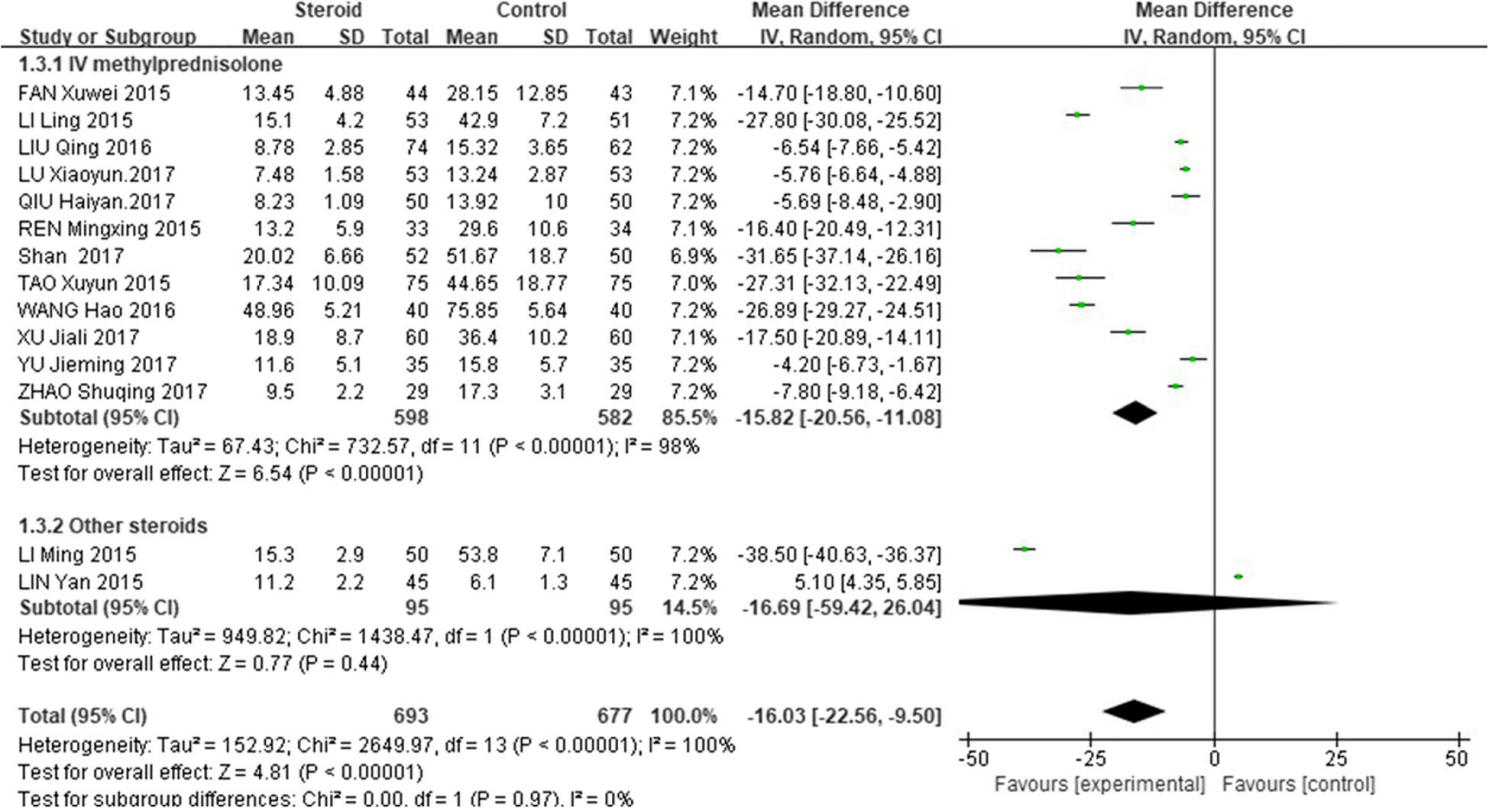
Forest plot for C-reactive protein level in subgroup analysis with use of methylprednisolone and other steroids

Use of glucocorticoids included the use of any type of glucocorticoids (e.g., methylprednisolone, dexamethasone, and prednisolone). The use of different types of glucocorticoids was different across studies. This might have contributed to the heterogeneity in the overall use of glucocorticoids. Thus, we stratified the meta-analysis by subgroup analyses. In subgroup meta-analysis for use of methylprednisolone compared with the use of other glucocorticoids for the length of hospital stay, the use of other steroids did not show any significant subgroup differences (Figs. 5, 6 and 7).

### Publication bias

**Fig. 8:**
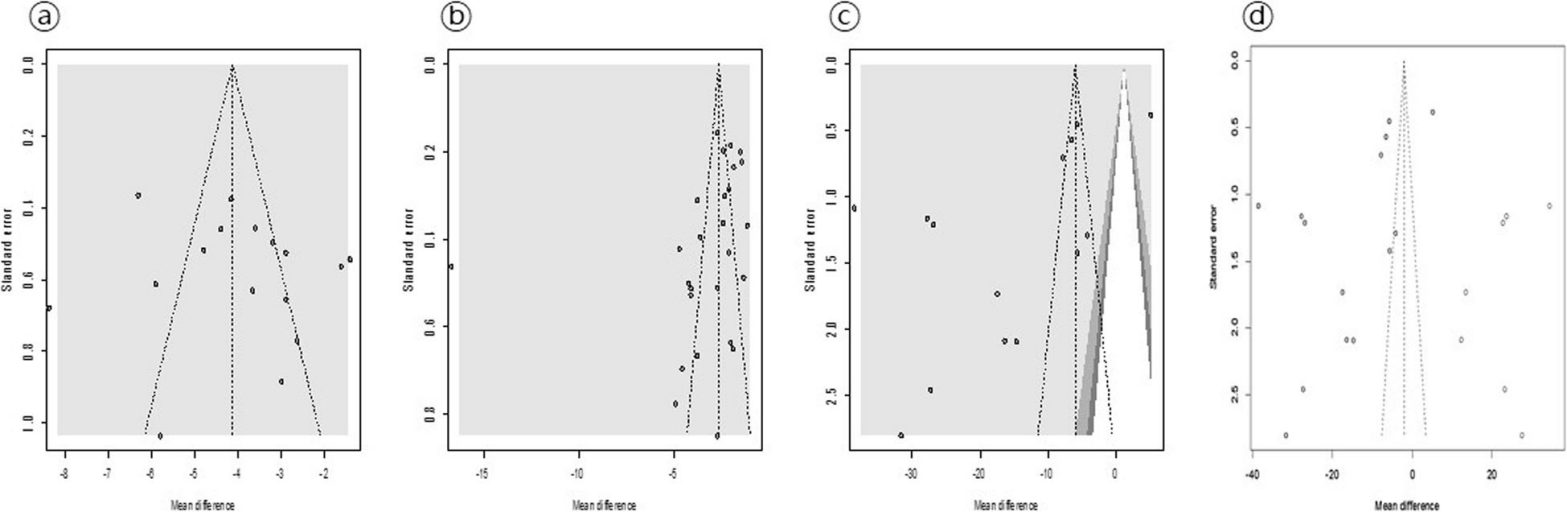
Funnel plots showing WMDs and 95% CIs for the efficacy of glucocorticoid therapy on the length of hospital stays (**a**), fever duration (**b**), and level of change of C reactive protein (**c**). Tests of asymmetry showed no significant deviation from the symmetry assumption (**a**, *P* = 0.56; **b**, *P* = 0.06). However, significant asymmetry was found in the level of change of CRP (**c**, *P* = 0.008). Trim-and-fill method for adjusting publication bias was used. Results showed substantial evidence of publication bias in CRP outcome (**d**)

All funnel plots were symmetric, indicating an absence of significant publication bias within these studies except for CRP outcome. Egger test results were − 1.73 (*P* = 0.09) for fever duration, 0.59 (*P* = 0.56) for hospital days, and − 3.19 (*P* = 0.008) for CRP. Trim-and-fill method for adjusting publication bias on CRP outcome was performed. The mean difference changed from – 3.27 (*P* = 0.35) to − 16.03 (*P* < 0.001). These results indicated that there was substantial evidence of publication bias in CRP outcome (Fig. 8).

## Discussion

This systematic review and meta-analysis identified and assessed RCTs on the use of glucocorticoids in children with MRMP. We specifically investigated effects of glucocorticoids on fever duration, length of hospital stay, and CRP level after treatment in comparison with conventional macrolide therapy. Results revealed positive effects of glucocorticoid treatment on all outcome measures.

*M. pneumoniae* is a common pathogen causing community acquired pneumonia. The clinical course of *M. pneumoniae* infection is diverse, ranging from self-limiting to severe pneumonia with extra-pulmonary complications [36]. Macrolide is considered the first-line treatment for *M. pneumoniae* infection [37]. In adults, one study reported that prednisone treatment for 7 days in patients with community-acquired pneumonia admitted to hospital can shorten time to clinical stability without increase in complications [38]. However, another study reported that glucocorticoid use did not show any benefits in children [8]. Pulmonary injury associated with severe mycoplasma pneumonia could be caused by host immune response rather than by direct microbial damage [39, 40]. Overly active cell-mediated immunity and cytokine responses play a significant role in MRMP [41]. Severe *M. pneumoniae* infections and MRMP show similar laboratory findings with severe acute respiratory syndrome such as increased levels of non-specific markers of inflammation such as serum CRP, lactate dehydrogenase, and D-dimer [42]. Since MRMP can be considered as an immune-mediated disease, use of immune modulatory therapy could seem rational. For cases with MRMP infection, secondary treatment options are limited due to adverse effects of tetracycline or fluoroquinolones, especially in children [43]. The use of systemic steroids, in addition to antimicrobial therapy, to diminish host immune response in MRMP has been used in children and demonstrated satisfactory effects [7, 8, 14]. Glucocorticoids have shown to improve clinical features and reduce lung injury in children and adults [5, 44, 45]. The use of glucocorticoids in *M. pneumoniae* infection showed reduced lung histopathologic score by reducing cytokines and decreasing inflammatory response ultimately leading to lower mortality [5, 44, 45]. Therefore, glucocorticoids have been considered suitable as adjunctive therapy to macrolide therapy. Although previous studies have reported the efficacy of glucocorticoids in the treatment of severe MRMP, treatment protocols varied from oral prednisolone of 1 mg/kg/day to intravenous methylprednisolone 30 mg/kg/day for 3 days [7, 17]. These variable treatment protocols are due to the lack of guidelines for glucocorticoids treatment in severe MRMP to date [7, 46].

In our study, the mean length of total duration of fever, mean length of hospital stay, and level of CRP after treatment in the glucocorticoid treatment group were significantly shorter or lower than that those in the conventional treatment group. However, heterogeneity in all outcome measures was high and sensitivity analysis resulted in no significant difference. Subgroup analysis showed no significant decrease in heterogeneity. In spite of high level of heterogeneity, all studies reported favorable outcomes of hospital stays and fever duration after the use of glucocorticoids in MRMP. For CRP level, only one study reported unfavorable result after the use of glucocorticoids in MRMP. Therefore, we could consider that the use of glucocorticoids has significantly favorable effects on outcomes of MRMP.

The strength of this review is that it summarized the current evidence on the efficacy of glucocorticoids from RCT. Conducting RCTs offers the potential to produce the most definitive evidence to confirm or refute whether glucocorticoids can help improve outcomes of MRMP.

This review has some limitations. Firstly, almost all studies included in this review had at least one methodological flaw. Secondly, publication bias was significant for change in CRP level. It might be due to small sample sizes in most studies and the fact that most studies were conducted in a single country. As a result, the robustness of these outcomes should be explored with future studies. Thirdly, most of the studies included were from a single country. Lastly, we could not evaluate other outcomes such as improvement in chest x-ray infiltration or other symptoms such as cough because these data were not available in each study.

Despite these limitations, with our review and meta-analysis, this is the first study to date to synthesize the efficacy of glucocorticoids in MRMP.

## Conclusions

In conclusion, even though some studies have reported the efficacy and effectiveness of systemic glucocorticoids in the treatment of MRMP [12–35], this is the first systematic review and meta-analysis to investigate the effectiveness of glucocorticoids in MRMP. We found that the use of glucocorticoids could shorten hospital days, shorten fever duration, and lower CRP levels after treatment.

However, these results should be interpreted cautiously, and future studies should also assess other outcomes to clarify the effect of glucocorticoids in MRMP.

## Supplementary information

**Additional file 1.** Search strategies for database searching

**Additional file 2.** PRISMA checklist

## Data Availability

The datasets used and/or analysed during the current study are available from the corresponding author on reasonable request.

## Abbreviations

CI: Confidence interval
CRP: C-reactive protein
*M. pneumoniae*: *Mycoplasma pneumoniae*
MRMP: Macrolide-refractory *M. pneumoniae*
PICO: Population-Intervention-Comparison-Outcome
PRISMA-P: Systematic Review and Meta-Analysis Protocols
RCT: Randomized control trial
WMD: weighted mean difference
