# Supplementary material for: Efficacy of glucocorticoids for the treatment of macrolide refractory mycoplasma pneumonia in children: meta-analysis of randomized controlled trials
Source: BMC Pulm Med. 2019 Dec 18;19:251. doi: 10.1186/s12890-019-0990-8 (PMC6921474; doi:10.1186/s12890-019-0990-8)
Supplement: Supplementary file 1 — Additional file 1. Search strategies for database searching [file 12890_2019_990_MOESM1_ESM.docx]

**Search strategies for database searching**

**Sources**

PubMed, EMBASE, and Cochrane were searched using separate comprehensive search strategies. In addition, database of core countries (Korea, China, and Japan) were searched using separate comprehensive search strategies. Database of core countries were as following;

- Korean database: KoreaMed (<https://koreamed.org>), National Digital Science Library (NDSL, <http://www.ndsl.kr>), Korean medical database (KMBASE, <http://kmbase.medric.or.kr/>), Research Information Sharing Service (RISS, <http://www.riss.kr>), Koreanstudies Information Service System (KISS, <http://kiss.kstudy.com/>)

- China: China National Knowledge Infrastructure (CNKI, <http://www.cnki.net>)

- Japan:, Japan Medical Abstracts Society, Igaku Chuo Zasshi (ICHUSHI, <http://www.jamas.or.jp>)

Search strategies

PubMed

| Number | Search query | results |
| --- | --- | --- |
| 1 | Mycoplasma pneumoniae[MeSH Terms] | 2758 |
| 2 | “Mycoplasma pneumoniae”[Title/Abstract] | 5294 |
| 3 | “M. pneumoniae”[Title/Abstract] | 2187 |
| 4 | #2 OR #3 | 5567 |
| 5 | #1 OR #4 | 5911 |
| 6 | Pneumonia, Mycoplasma[MeSH Terms] | 3603 |
| 7 | “Mycoplasma pneumonia”[Title/Abstract] | 576 |
| 8 | “Primary Atypical Pneumonia”[Tile/Abstract] | 315 |
| 9 | “Mycoplasma pneumoniae pneumonia”[Title/Abstract] | 366 |
| 10 | “Mycoplasma pneumonias”[Title/Abstract] | 15 |
| 11 | #7 OR #8 OR #9 OR #10 | 1231 |
| 12 | #6 OR #11 | 4069 |
| 13 | #5 OR #12 | 7430 |
| 14 | Anti-Inflammatory Agents[MeSH Terms] | 139370 |
| 15 | Antiinflammatories[Title/Abstract] | 97 |
| 16 | “Anti-Inflammatories”[Title/Abstract] | 768 |
| 17 | “Antiinflammatory Agents”[Title/Abstract] | 1130 |
| 18 | “Anti-Inflammatory Agents”[Title/Abstract] | 6523 |
| 19 | #15 OR #16 OR #17 OR #18 | 8454 |
| 20 | #14 OR #19 | 143360 |
| 21 | Steroids[MeSH Terms] | 794971 |
| 22 | Steroid*[Title/Abstract] | 212103 |
| 23 | #21 or #22 | 905218 |
| 24 | Glucocorticoids[MeSH Terms] | 56432 |
| 25 | Glucocorticoid*[Title/Abstract] | 59965 |
| 26 | #24 OR #25 | 96605 |
| 27 | Prednisolone[MeSH Terms] | 47756 |
| 28 | Prednisolone*[Title/Abstract] | 24830 |
| 29 | #27 OR #28 | 58223 |
| 30 | Prednisone[MeSH Terms] | 37085 |
| 31 | Prednisone*[Title/Abstract] | 26016 |
| 32 | #30 OR #31 | 49160 |
| 33 | Methylprednisolone[MeSH Terms] | 17962 |
| 34 | Methylprednisolone *[Title/Abstract] | 14137 |
| 35 | Methyl-prednisolone *[Title/Abstract] | 894 |
| 36 | #34 OR #35 | 14927 |
| 37 | #33 OR #36 | 24162 |
| 38 | Methylprednisolone Hemisuccinate[MeSH Terms] | 687 |
| 39 | Solumedrol[Title/Abstract] | 114 |
| 40 | “Solu-medrol”[Title/Abstract] | 119 |
| 41 | #39 OR #40 | 233 |
| 42 | #38 OR #41 | 860 |
| 43 | Adrenal cortex hormones[MeSH Terms] | 264003 |
| 44 | Adrenal cortex hormones*[Title/Abstract] | 10505 |
| 45 | #43 OR #44 | 264121 |
| 46 | Hydrocortisone[MeSH Terms] | 68365 |
| 47 | Hydrocortisone*[Title/Abstract] | 17537 |
| 48 | #46 OR #47 | 73016 |
| 49 | #20 OR #23 OR #26 OR #29 OR # 32 OR #37 OR #42 OR #45 OR #48 | 1097699 |
| 50 | #13 AND #49 | 296 |
| 51 | (((((Mycoplasma pneumoniae[MeSH Terms]) OR ((“Mycoplasma pneumoniae”[Title/Abstract]) OR Mycoplasma pneumoniae[MeSH Terms]))) OR ((Pneumonia, Mycoplasma[MeSH Terms]) OR ((((“Mycoplasma pneumonia”[Title/Abstract]) OR “Primary Atypical Pneumonia”[Tile/Abstract]) OR “Mycoplasma pneumoniae pneumonia”[Title/Abstract]) OR “Mycoplasma pneumonias”[Title/Abstract])))) AND (((((((((((Anti-Inflammatory Agents[MeSH Terms]) OR ((((Antiinflammatories[Title/Abstract]) OR “Anti-Inflammatories”[Title/Abstract]) OR “Antiinflammatory Agents”[Title/Abstract]) OR “Anti-Inflammatory Agents”[Title/Abstract]))) OR ((Steroids[MeSH Terms]) OR Steroid*[Title/Abstract])) OR ((Glucocorticoids[MeSH Terms]) OR Glucocorticoid*[Title/Abstract])) OR ((Prednisolone[MeSH Terms]) OR Prednisolone*[Title/Abstract])) OR ((Prednisone[MeSH Terms]) OR Prednisone*[Title/Abstract])) OR ((Methylprednisolone[MeSH Terms]) OR ((Methylprednisolone *[Title/Abstract]) OR Methyl-prednisolone *[Title/Abstract]))) OR ((Methylprednisolone Hemisuccinate[MeSH Terms]) OR ((Solumedrol[Title/Abstract]) OR “Solu-medrol”[Title/Abstract]))) OR ((Adrenal cortex hormones[MeSH Terms]) OR Adrenal cortex hormones*[Title/Abstract])) OR ((Hydrocortisone[MeSH Terms]) OR Hydrocortisone*[Title/Abstract])) | 296 |

EMBASE

| Number | Search query | results |
| --- | --- | --- |
| 1 | ‘Mycoplasma pneumoniae’/exp | 6,614 |
| 2 | ‘Mycoplasma pneumoniae’:ab,ti | 6,393 |
| 3 | #1 OR #2 | 8,555 |
| 4 | ‘M. pneumoniae’:ab,ti | 2,575 |
| 5 | ‘Mycoplasma pneumonia’/exp | 3,755 |
| 6 | ‘Mycoplasma pneumonia’:ab,ti | 805 |
| 7 | ‘Primary atypical pneumonia’:ab,ti | 310 |
| 8 | ‘Mycoplasma pneumoniae pneumonia’:ab,ti | 438 |
| 9 | #6 OR #7 OR #8 | 1,510 |
| 10 | #5 OR #9 | 4,317 |
| 11 | ‘Mycoplasma pneumonias’:ab,ti | 20 |
| 12 | #3 OR #4 OR #10 OR #11 | 10,633 |
| 13 | antiinflammatories:ab,ti | 199 |
| 14 | 'anti-inflammatories':ab,ti | 1,128 |
| 15 | antiinflammatory agent'/exp | 1,683,729 |
| 16 | 'antiinflammatory agents':ab,ti | 1,921 |
| 17 | 'anti-inflammatory agents':ab,ti | 7,624 |
| 18 | #16 OR #17 | 9,484 |
| 19 | #15 OR #18 | 1,685,471 |
| 20 | 'steroid'/exp | 1,389,799 |
| 21 | steroid*:ab,ti | 283,695 |
| 22 | #20 OR #21 | 1,468,138 |
| 23 | 'glucocorticoid'/exp | 658,094 |
| 24 | glucocorticoid*:ab,ti | 75,285 |
| 25 | #23 OR #24 | 676,327 |
| 26 | 'prednisolone'/exp | 113,687 |
| 27 | prednisolone*:ab,ti | 32,939 |
| 28 | #26 OR #27 | 119,790 |
| 29 | 'prednisone'/exp | 155,510 |
| 30 | prednisone*:ab,ti | 39,232 |
| 31 | #29 OR #30 | 160,522 |
| 32 | 'methylprednisolone'/exp | 81,253 |
| 33 | methylprednisolone*:ab,ti | 20,573 |
| 34 | 'methyl-prednisolone*':ab,ti | 1,510 |
| 35 | #33 OR #34 | 21,882 |
| 36 | #32 OR #35 | 84,786 |
| 37 | 'methylprednisolone sodium succinate'/exp | 6,315 |
| 38 | solumedrol:ab,ti | 488 |
| 39 | 'solu-medrol':ab,ti | 199 |
| 40 | #38 OR #39 | 683 |
| 41 | #37 OR #40 | 6,410 |
| 42 | 'corticosteroid'/exp | 863,033 |
| 43 | 'adrenal cortex hormone*':ab,ti | 611 |
| 44 | #42 OR #43 | 863,087 |
| 45 | 'hydrocortisone'/exp | 120,575 |
| 46 | hydrocortisone*:ab,ti | 19,790 |
| 47 | #45 OR #46 | 124,603 |
| 48 | #13 OR #14 OR #19 OR #22 OR #25 OR #28 OR #31 OR #36 OR #41 OR #44 OR #47 | 2,434,793 |
| 49 | #12 AND #48 | 1,564 |

Cochrane

| Number | Search query | results |
| --- | --- | --- |
| 1 | MeSH descriptor: [Mycoplasma pneumonia] explode all trees | 24 |
| 2 | “Mycoplasma pneumoniae”:ab,ti | 113 |
| 3 | “M. pneumoniae”:ab,ti | 43 |
| 4 | #2 OR #3 | 117 |
| 5 | #1 OR #4 | 120 |
| 6 | MeSH descriptor: [Pneumonia, Mycoplasma] explode all trees | 40 |
| 7 | “Mycoplasma pneumonia”:ab,ti | 21 |
| 8 | “Primary atypical pneumonia”:ab,ti | 2 |
| 9 | “Mycoplasma pneumoniae pneumonia”:ab,ti | 14 |
| 10 | “Mycoplasma pneumonia”:ab,ti | 0 |
| 11 | #7 OR #8 OR #9 OR #10 | 35 |
| 12 | #6 OR #11 | 65 |
| 13 | #5 OR #12 | 145 |
| 14 | MeSH descriptor: [Anti-Inflammatory Agents] explode all trees | 12500 |
| 15 | antiinflammatories:ab,ti | 6 |
| 16 | “anti-inflammatories”:ab,ti | 63 |
| 17 | “antiinflammatory agents”:ab,ti | 64 |
| 18 | “anti-inflammatory agents”:ab,ti | 332 |
| 19 | #15 OR #16 OR #17 OR #18 | 461 |
| 20 | #14 OR #19 | 12809 |
| 21 | MeSH descriptor: [Steroids] explode all trees | 44003 |
| 22 | steroid*:ab,ti | 15216 |
| 23 | #21 or #22 | 54508 |
| 24 | MeSH descriptor: [Glucocorticoids] explode all trees | 4091 |
| 25 | Glucocorticoid*:ab,ti | 2817 |
| 26 | #24 OR #25 | 6096 |
| 27 | MeSH descriptor: [Prednisolone] explode all trees | 3602 |
| 28 | prednisolone*:ab,ti | 3328 |
| 29 | #27 OR #28 | 5513 |
| 30 | MeSH descriptor: [Prednisone] explode all trees | 2944 |
| 31 | prednisone*:ab,ti | 4837 |
| 32 | #30 OR #31 | 5838 |
| 33 | MeSH descriptor: [Methylprednisone] explode all trees | 1702 |
| 34 | methylprednisolone*:ab,ti | 2293 |
| 35 | “methyl-prednisolone*”:ab,ti | 133 |
| 36 | #34 OR #35 | 2397 |
| 37 | #33 OR #36 | 2924 |
| 38 | MeSH descriptor: [Methylprednisone Hemisuccinate] explode all trees | 56 |
| 39 | solumedrol:ab,ti | 19 |
| 40 | “solu-medrol”:ab,ti | 27 |
| 41 | #39 OR #40 | 45 |
| 42 | #38 OR #41 | 92 |
| 43 | MeSH descriptor: [Adrenal Cortex Hormones] explode all trees | 13079 |
| 44 | “adrenal cortex hormone*”:ab,ti | 5 |
| 45 | #43 OR #44 | 13083 |
| 46 | MeSH descriptor: [Hydrocortisone] explode all trees | 5170 |
| 47 | hydrocortisone*:ab,ti | 1639 |
| 48 | #46 OR #47 | 6024 |
| 49 | #20 OR #23 OR #26 OR #29 OR #32 OR #37 OR #42 OR #45 OR #48 | 69866 |
| 50 | #13 OR #49 | 11 |

Korea database

| Database | Search query | results |
| --- | --- | --- |
| NDSL | (("mycoplasma pneumoniae" AND "Antiinflammatory Agents") OR ("mycoplasma pneumoniae" AND "Anti-Inflammatory Agents") OR ("mycoplasma pneumoniae" AND Steroid*) OR ("mycoplasma pneumoniae" AND Glucocorticoid*) OR ("mycoplasma pneumoniae" AND Prednisolone*) OR ("mycoplasma pneumoniae" AND Prednisone*) OR "mycoplasma pneumoniae" AND Methylprednisolone*) OR ("mycoplasma pneumoniae" AND Solumedrol) OR ("mycoplasma pneumoniae" AND Adrenal cortex hormone*) OR ("mycoplasma pneumoniae" AND Hydrocortisone*) OR ("mycoplasma pneumonia" AND "Antiinflammatory Agents") OR ("mycoplasma pneumonia" AND "Anti-Inflammatory Agents") OR ("mycoplasma pneumonia" AND Steroid*) OR ("mycoplasma pneumonia" AND Glucocorticoid*) OR ("mycoplasma pneumonia" AND Prednisolone*) OR ("mycoplasma pneumonia" AND Prednisone*) OR ("mycoplasma pneumonia" AND Methylprednisolone*) OR ("mycoplasma pneumonia" AND Solumedrol) OR ("mycoplasma pneumonia" AND Adrenal cortex hormone*) OR ("mycoplasma pneumonia" AND Hydrocortisone*) OR ("Mycoplasma pneumoniae pneumonia" AND "Antiinflammatory Agents") OR ("Mycoplasma pneumoniae pneumonia" AND "Anti-Inflammatory Agents") OR ("Mycoplasma pneumoniae pneumonia" AND Steroid*) OR ("Mycoplasma pneumoniae pneumonia" AND Glucocorticoid*) OR ("Mycoplasma pneumoniae pneumonia" AND Prednisolone*) OR ("Mycoplasma pneumoniae pneumonia" AND Prednisone*) OR ("Mycoplasma pneumoniae pneumonia" AND Methylprednisolone*) OR ("Mycoplasma pneumoniae pneumonia" AND Solumedrol) OR ("Mycoplasma pneumoniae pneumonia" AND Adrenal cortex hormone*) OR ("Mycoplasma pneumoniae pneumonia" AND Hydrocortisone*) OR (“Pneumonia, Mycoplasma“ AND "Antiinflammatory Agents") OR (“Pneumonia, Mycoplasma“ AND "Anti-Inflammatory Agents") OR (“Pneumonia, Mycoplasma“ AND Steroid*) OR (“Pneumonia, Mycoplasma“ AND Glucocorticoid*) OR (“Pneumonia, Mycoplasma“ AND Prednisolone*) OR (“Pneumonia, Mycoplasma“ AND Prednisone*) OR (“Pneumonia, Mycoplasma“ AND Methylprednisolone*) OR (“Pneumonia, Mycoplasma“ AND Solumedrol) OR (“Pneumonia, Mycoplasma“ AND Adrenal cortex hormone*) OR (“Pneumonia, Mycoplasma“ AND Hydrocortisone*)) | 11 |
| KMBASE | ((((([ALL=mycoplasma pneumoniae] OR [ALL=M. pneumoniae]) OR [ALL=mycoplasma pneumonia])) OR [ALL=Primary Atypical Pneumonia]) OR [ALL=Mycoplasma pneumoniae pneumonia]) OR [ALL=Mycoplasma Pneumonias]) AND (((((((([ALL=Anti-Inflammatory Agent*] OR [ALL=Steroid*]) OR [ALL=Glucocorticoid*]) OR [ALL=Prednisolone*]) OR [ALL=Prednisone*]) OR [ALL=Methylprednisolone*]) OR [ALL=Methylprednisolone Hemisuccinate*]) OR [ALL=Adrenal cortex hormone*]) OR [ALL=Hydrocortisone*]) | 19 |
| KoreaMed | ("mycoplasma pneumoniae" [ALL] OR "M. pneumoniae" [ALL] OR "mycoplasma pneumonia" [ALL] OR "Primary Atypical Pneumonia" [ALL] OR "Mycoplasma pneumoniae pneumonia" [ALL] OR "Mycoplasma Pneumonias" [ALL] ) AND ( "Anti-Inflammatory Agent*" [ALL] OR Steroid* [ALL] OR Glucocorticoid* [ALL] OR Prednisolone* [ALL] OR Prednisone* [ALL] OR Methylprednisolone* [ALL] OR Methylprednisolone Hemisuccinate* [ALL] OR Adrenal cortex hormone* [ALL] OR Hydrocortisone* [ALL] ) | 1 |
| RISS | (("mycoplasma pneumoniae" AND Antiinflammatories) OR ("mycoplasma pneumoniae" AND Steroid*) OR ("mycoplasma pneumoniae" AND Glucocorticoid*) OR ("mycoplasma pneumoniae" AND Prednisolone*) OR (mycoplasma pneumoniae" AND Prednisone*) OR ("mycoplasma pneumoniae" AND Methylprednisolone*) OR ("mycoplasma pneumoniae" AND Methyl-prednisolone*) OR "mycoplasma pneumoniae" AND Solumedrol) OR ("mycoplasma pneumoniae" AND Adrenal cortex hormone*) OR (mycoplasma pneumoniae" AND Hydrocortisone*) OR (mycoplasma pneumonia" AND Antiinflammatories) OR (mycoplasma pneumonia" AND Steroid*) OR ("mycoplasma pneumonia" AND Glucocorticoid*) OR ("mycoplasma pneumonia" AND Prednisolone*) OR ("mycoplasma pneumonia" AND Prednisone*) OR ("mycoplasma pneumonia" AND Methylprednisolone*) OR ("mycoplasma pneumonia" AND Methyl-prednisolone*) OR ("mycoplasma pneumonia" AND Solumedrol) OR ("mycoplasma pneumonia" AND Adrenal cortex hormone*) OR ("mycoplasma pneumonia" AND Hydrocortisone*) OR ("M. pneumoniae" AND Antiinflammatories) OR ("M. pneumoniae" AND Steroid*) OR ("M. pneumoniae" AND Glucocorticoid*) OR ("M. pneumoniae" AND Prednisolone*) OR ("M. pneumoniae" AND Prednisone*) OR ("M. pneumoniae" AND Methylprednisolone*) OR ("M. pneumoniae" AND Methyl-prednisolone*) OR ("M. pneumoniae" AND Solumedrol) OR ("M. pneumoniae" AND Adrenal cortex hormone*) OR ("M. pneumoniae" AND Hydrocortisone*)) | 20 |
| KISS | (("mycoplasma pneumoniae" AND Antiinflammatories) OR ("mycoplasma pneumoniae" AND Steroid*) OR ("mycoplasma pneumoniae" AND Glucocorticoid*) OR ("mycoplasma pneumoniae" AND Prednisolone*) OR (mycoplasma pneumoniae" AND Prednisone*) OR ("mycoplasma pneumoniae" AND Methylprednisolone*) OR ("mycoplasma pneumoniae" AND Methyl-prednisolone*) OR "mycoplasma pneumoniae" AND Solumedrol) OR ("mycoplasma pneumoniae" AND Adrenal cortex hormone*) OR (mycoplasma pneumoniae" AND Hydrocortisone*) OR (mycoplasma pneumonia" AND Antiinflammatories) OR (mycoplasma pneumonia" AND Steroid*) OR ("mycoplasma pneumonia" AND Glucocorticoid*) OR ("mycoplasma pneumonia" AND Prednisolone*) OR ("mycoplasma pneumonia" AND Prednisone*) OR ("mycoplasma pneumonia" AND Methylprednisolone*) OR ("mycoplasma pneumonia" AND Methyl-prednisolone*) OR ("mycoplasma pneumonia" AND Solumedrol) OR ("mycoplasma pneumonia" AND Adrenal cortex hormone*) OR ("mycoplasma pneumonia" AND Hydrocortisone*) OR ("M. pneumoniae" AND Antiinflammatories) OR ("M. pneumoniae" AND Steroid*) OR ("M. pneumoniae" AND Glucocorticoid*) OR ("M. pneumoniae" AND Prednisolone*) OR ("M. pneumoniae" AND Prednisone*) OR ("M. pneumoniae" AND Methylprednisolone*) OR ("M. pneumoniae" AND Methyl-prednisolone*) OR ("M. pneumoniae" AND Solumedrol) OR ("M. pneumoniae" AND Adrenal cortex hormone*) OR ("M. pneumoniae" AND Hydrocortisone*)) | 14 |

Japan Medical Abstracts Society, Igaku Chuo Zasshi (ICHUSHI) <http://www.jamas.or.jp>

| Number | Search query | results |
| --- | --- | --- |
| 1 | “Mycoplasma pneumoniae”/AL | 75 |
| 2 | ”M. pneumoniae”/AL | 0 |
| 3 | “Mycoplasma pneumonia”/AL | 78 |
| 4 | “Primary Atypical Pneumonia”/AL | 0 |
| 5 | “Mycoplasma pneumoniae pneumonia”/AL | 1 |
| 6 | “Mycoplasma pneumonias”/AL | 0 |
| 7 | #1 or #2 or #3 or #4 or #5 or #6 | 78 |
| 8 | Antiinflammatories/AL | 0 |
| 9 | “Anti-inflammatories”/AL | 0 |
| 10 | “Antiinflammatory Agents”/AL | 0 |
| 11 | “Anti-Inflammatory Agents”/AL | 1 |
| 12 | Steroid/AL | 1251 |
| 13 | Glucocorticoid/AL | 174 |
| 14 | Prednisolone/AL | 1831 |
| 15 | Prednisone/AL | 29 |
| 16 | Methylprednisolone/AL | 652 |
| 17 | “Methyl-prednisolone”/AL | 0 |
| 18 | Solumedrol/AL | 0 |
| 19 | “Solu-medrol”/AL | 0 |
| 20 | “Adrenal cortex hormone”/AL | 0 |
| 21 | Hydrocortisone/AL | 225 |
| 22 | #8 or #9 or #10 or #11 or #12 or #13 or #14 or #15 or #16 or #17 or #18 or #19 or #20 or #21 | 3410 |
| 23 | #7 and # 22 | 4 |
| 24 | ((“Mycoplasma pneumoniae”/AL) or (”M. pneumoniae”/AL) or (“Mycoplasma pneumonia”/AL) or (“Primary Atypical Pneumonia”/AL) or (“Mycoplasma pneumoniae pneumonia”/AL) or (“Mycoplasma pneumonias”/AL)) and ((Antiinflammatories/AL) or (“Anti-inflammatories”/AL) or (“Antiinflammatory Agents”/AL) or (“Anti-Inflammatory Agents”/AL) or (Steroid/AL Glucocorticoid/AL) or (Prednisolone/AL) or (Prednisone/AL) or (Methylprednisolone/AL) or (“Methyl-prednisolone”/AL) or (Solumedrol/AL) or (“Solu-medrol”/AL) or (“Adrenal cortex hormone”/AL) or (Hydrocortisone/AL)) | 4 |

China National Knowledge Infrastructure (CNKI) <http://www.cnki.net>

| Number | Search query | results |
| --- | --- | --- |
| 1 | (((TI = “mycoplasma pneumoniae” OR AB = “mycoplasma pneumoniae”) OR (TI = M. pneumoniae” OR AB = “M pneumoniae”) OR (TI = “mycoplasma pneumonia” OR AB = “mycoplasma pneumonia”) OR (((TI = “Primary Atypical Pneumonia” OR AB = “Primary Atypical Pneumonia”) OR (TI = “mycoplasma pneumoniae pneumonia” OR AB = “mycoplasma pneumoniae pneumonia”) OR (TI = “mycoplasma pneumonias” OR AB = “mycoplasma pneumonias”)) AND ((TI = “Anti-Inflammatories” OR AB = “Anti-Inflammatories”) or (TI = “Antiinflammatory agents” OR AB = “Antiinflammatory agents”“) or (TI = “Anti-Inflammatory agents” OR AB = “Anti-Inflammatory agents”“) OR (TI = Steroid OR AB = Steroid) OR (TI = Glucocorticoid OR AB = Glucocorticoid) OR (TI = Prednisolone OR AB = Prednisolone) OR (TI = Prednisone OR AB = Prednisone) OR (TI = Methylprednisolone OR AB = Methylprednisolone) OR (TI = Methyl-prednisolone OR AB = Methyl-prednisolone) OR (TI = Solumedrol OR AB = Solumedrol) OR (TI = Solu-medrol OR AB = Solu-medrol) OR (TI = “Adrenal cortex hormone” OR AB = “Adrenal cortex hormone”) OR (TI = Hydrocortisone OR AB = Hydrocortisone))) | 202 |
